# Monitoring and analysis of coastal reclamation from 1995–2015 in Tianjin Binhai New Area, China

**DOI:** 10.1038/s41598-017-04155-0

**Published:** 2017-06-20

**Authors:** Wengang Chen, Dongchuan Wang, Yong Huang, Liding Chen, Lihui Zhang, Xiangwang Wei, Mengqin Sang, Feicui Wang, Jinya Liu, Bingxu Hu

**Affiliations:** 1 School of Geology and Geomatics, Tianjin Chengjian University, No. 26 Jinjing RD, Xiqing District, Tianjin, 300384 China; 2 State Key Lab of Urban and Regional Ecology, Research Center for Eco-Environmental Science, Chinese Academy of Sciences, Beijing, 100101 China; 3 Research and Development Center, China Academy of Launch Vehicle Technology, No. 1 Nan Dahongmen Road, Fengtai District, Beijing, 100076 China

## Abstract

Increasing coastal reclamation activities have been undertaken to solve the conflict between people and land resources, creating significant challenges for coordinating coastal reclamation, economic development and environmental protection. This paper analyzes the effects of coast reclamation on Gross Domestic Product growth and the quality of inshore seawater in the Tianjin Binhai New Area. Remote sensing and a Geographic Information System were used to extract the information of coastal reclamation. The correlation between the area of coastal reclamation, GDP growth and the quality of inshore seawater was analyzed and a decoupling elasticity model was used to explore trends in the relationship between economic development and coastal reclamation. Results showed that coastal reclamation activities played an important role in promoting economic development, but greatly damaged the ecological environment. Although the relationship between coastal reclamation and economic development has weakened during the last three periods, the influence on the environment will continue because of the cumulative effects of pollution. To maintain a balance between coastal reclamation, economic development and environmental protection, (1) coastal reclamation planning must address both economic and environmental outcomes; (2) environmental deficiencies from existing coastal reclamation projects must be rectified; and (3) the legal system regulating coastal reclamation needs to be refined and strengthened.

## Introduction

Driven by an increasing need for land resources, more and more reclamation activities are occurring in coastal areas around the world. Human activities have become one of the main drivers of coastline changes^1–3^, exceeding the influence of natural factors such as storm surges, tides, sediment supply, land subsidence and global climate change^4–6^. In China, rapid economic growth and urbanization have significantly expanded the area of coastal reclamation^7, 8^. Extensive coastal reclamation activities are, in turn, boosting rapid economic development in China’s coastal economy, and China’s economy growth is increasingly concentrated in the coastal region^9–11^. The 11 coastal provinces or metropolises, which cover 13% of China and host 43.5% of the nation’s population, contributed 60.8% of the national GDP in 2011^8^.

Although land reclamation results in significant benefits to society, it causes serious environmental problems. Coastal wetlands in China are now under severe pressure and at great potential risk^8^. The rapid expansion of artificial coastlines to seaward creates irreversible changes to the natural coastline^9^ and reduces the hydrodynamic condition and pollutant diffusion ability of offshore marine areas, leading to the deterioration of water quality and the loss of marine biodiversity^12–14^. The decline in the marine ecological environment^11^, which lowers the economic value of ecosystem services, poses a potentially hazard to human life and assets^15, 16^, thus cutting down the benefits human populations derive, directly or indirectly, from nature^17, 18^. Satisfactory management of coastal land reclamation is a challenge facing governments in China^11^.

The effects of coastal reclamation on marine ecological environments and biodiversity have been widely analyzed. Priyandes and Majid^19^ studied the impact of reclamation activities on the northern coast of Batam Island. Results showed that reclamation activities changed the coastal morphology and hydro-oceanography and led to the deterioration of mangroves and coral reefs. Wang *et al*.^20^ used a high-resolution numerical modeling method to assess the cumulative effect of coastal projects on hydrodynamic conditions, showing that the tidal current speed and tidal prism have decreased by 40% in the western part of the bay and 20% in the eastern part of the bay from 1938 to 2007 in the full-tide area. Wang *et al*.^21^ examined spatial and temporal variation in soil organic carbon (SOC) and soil total nitrogen (STN) in a coastal reclamation area in eastern China and revealed that the reclamation area from the tidal flat should be a potential sink for SOC and STN. Feng *et al*.^22^ evaluated coastal reclamation feasibility in Hangzhou Bay and concluded that hydrodynamic conditions are important environmental factors in coastal zones, determining the water exchange capacity and directly related to water quality. Shen *et al*.^23^ studied the cumulative impact of reclamations on coastal ecosystem health and concluded that coastal reclamations have a historically cumulative detrimental effect on the benthic environment. Bulleri and Chapman’s study^24^ showed that the introduction of urban infrastructure in the intertidal zone or in near shore waters can cause fragmentation and loss of natural habitats. Perkins *et al*.^25^ reviewed the nature of ecological impacts of coastal structures, finding that coastal structures alter important physical, chemical and biological processes of inter tidal habitats and strongly impact community structure, inter-habitat linkages and ecosystem services while also driving habitat loss.

These researches highlighted some of the problems relevant to the planning and management of coastal reclamation. However, information on the rapid growth of artificial coastlines and the relationship between coastal reclamation and the ecological environment is inadequate^25^. Some works^26–28^ have analyzed the changes in coastlines and their effects on the ecological environment. This is of great significance for the planning and management of coastal reclamation and the coordinated development of coastal reclamation and sea water resources protection. However, coordinated development relies on a balance of coastal reclamation, economic development and environmental protection. It goes against the concept of sustainable development to enhance economic development by sacrificing the ecological environment in the process of coastal reclamation. Equally, it is clearly undesirable to deny all coastal reclamation activities just for environmental protection. Therefore, both economic development and environmental protection should be included in the process of coastal reclamation.

This paper analyzes the effects of coast reclamation on GDP growth and the quality of inshore seawater from 1995 to 2015, using the Tianjin Binhai New Area (TBNA) as the study area. Remote sensing (RS) and geographical information system (GIS) were used to measure the changes in the length of coastline and the area of coastal reclamation in TBNA. The reclaimed lands were extracted from RS images and divided into two classes, construction land (being used for construction) and wetland (being in the process of reclamation and still covered with water). A correlation analysis between the area of coastal reclamation, GDP growth and the quality of inshore seawater was carried out and a decoupling elasticity model was used to explore the trend of the relationship between economic development and coastal reclamation.

## Results

### Coastline changes in the TBNA

**Figure 1:**
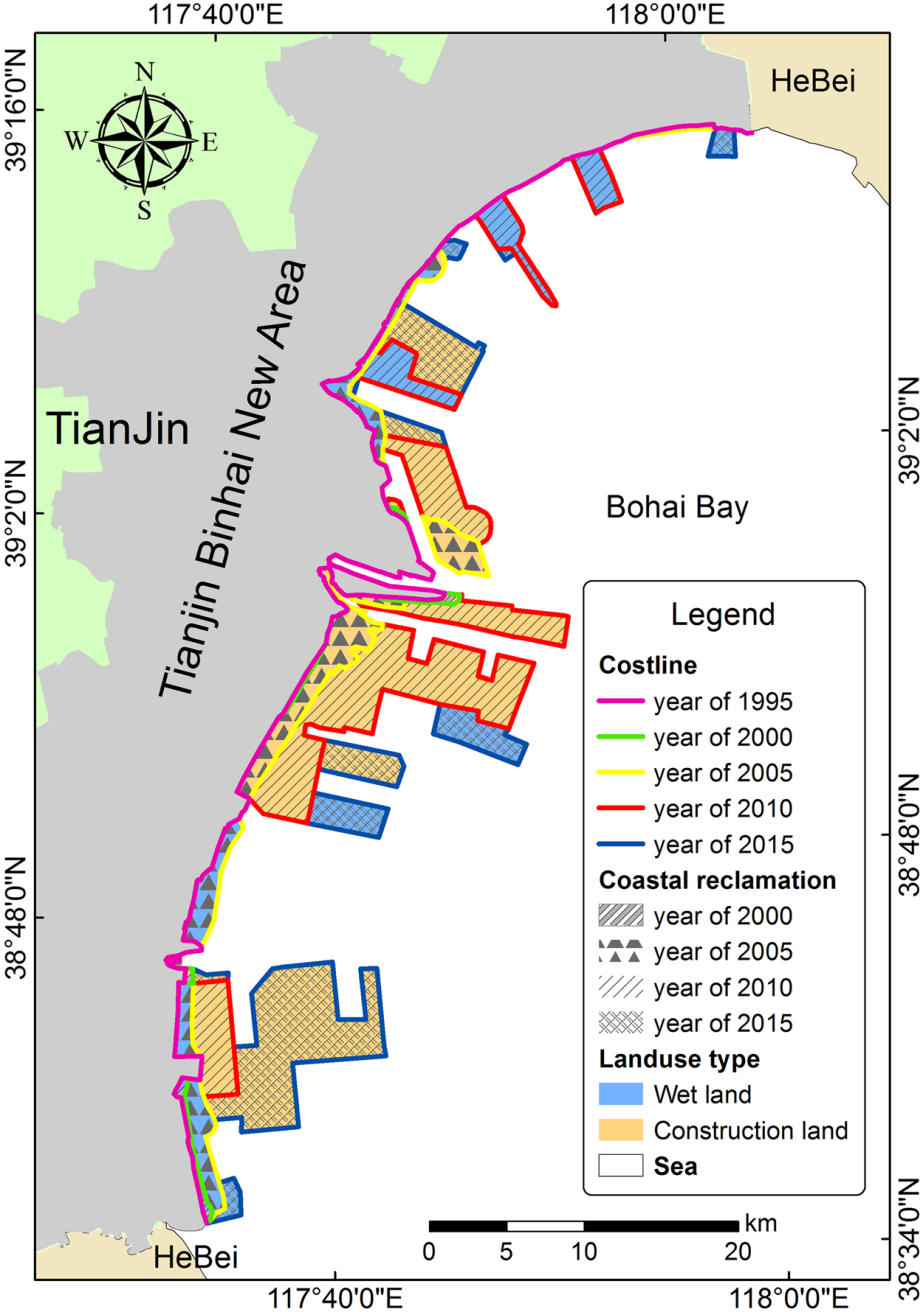
Coastline changes for the TBNA from1995 to 2015. Figure 1 was generated by ArcGIS v10.2 software (Environmental Systems Research Institute, Inc, USA, URL http://www.esri.com/).

Coastline data in 1995, 2000, 2005, 2010 and 2015 were interpreted (Fig. 1), and the length of coastline and coastal reclamation area in different periods were calculated (Table 1).

**Table 1:** Statistics for coastline length and coastal reclamation area in the TBNA.

Year	Cumulative coastline length(km)	Coastal reclamation area(5-yearly summed in km^2^)	
		Wet land	Construction land
1995	134.42	—	—
2000	133.98	3.74	2.52
2005	145.48	36.49	32.09
2010	250.38	26.84	127.67
2015	319.24	28.62	99.38

From Fig. 1 and Table 1, it can be inferred that the cumulative length of coastline in the TBNA increased overall, and the total increase during the 20 year period was 184.82 km. The main types of coastal reclamation were wetland and construction land. From 1995 to 2000, the 5-yearly summed area of coastal reclamation increased while the length of coastline slightly decreased because some indentations in the bay were filled by the artificial coastline. Coastal reclamation occurred mainly in the Nangang industrial zone, the Harbor industrial zone, and part of Beijiang port. From 2000 to 2005, the length of coastline increased by 11.5 km: the wetland area increased by 36.49 km^2^ and construction land area by 32.09 km^2^. Coastal reclamation during this period mainly occurred in the Nangang industrial zone, the Harbor industrial zone, Nanjiang port, Beijiang port, Dongjiang port and the Binhai leisure zone, covering a large region but with a relatively small area of change. From 2005 to 2010, the highest change rate of coastline was measured and the coastline increment reached the maximum across the whole time series. The area of reclaimed construction land rose sharply, increasing by 127.67 km^2^ and the area of wetland increased by 26.84 km^2^, about 10 km^2^ less than in the last period. Construction land in coastal reclamation mainly occurred in Dongjiang port, Nanjiang port, Beijiang port, the Harbor industrial zone and the Nangang industrial zone while wetlands occurred mainly in the area of the Central fishing port and Binhai leisure zone. From 2010 to 2015, the length of coastline increased by 68.86 km and the change rate of the coastline declined compared with the last period, although the increase was still high. During this period, construction land in coastal reclamation increased by 99.38 km^2^. The reclamation of construction land mainly occurred in the Nangang industrial zone, part of Binhai leisure zone and the Harbor industrial zone. Wetland reclamation occurred mainly in the Harbor industrial zone, Central fishing port and parts of the Nangang industrial zone.

With the launching of a series of national policies, coastal reclamation activities in the TBNA form part of a long-term plan. With a total investment of CNY 60 billion until 2018, it is expected that the total area of coastal reclamation will reach about 200 km^2^.

### Results of correlation analysis

**Figure 2:**
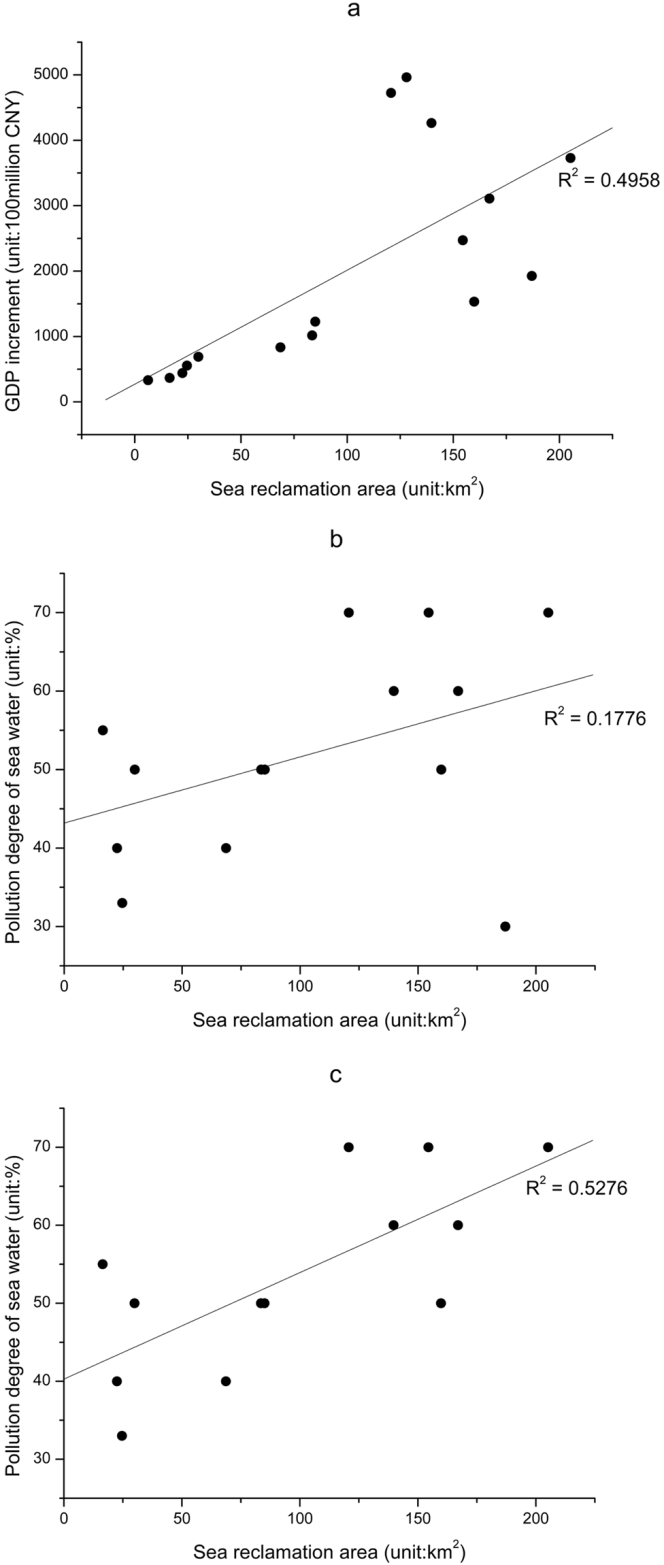
Scatter diagrams. (**a**) Coastal reclamation area and GDP growth, (**b**) Coastal reclamation area and pollution degree of sea water, and (**c**) Coastal reclamation area and pollution degree of sea water except for 2009.

National policy plays an important role in promoting coastline changes. The natural coastline influenced by human activities shows irreversible changes, which have a severe impact on the marine ecological environment. Therefore, it is necessary to analyze the relationship between coastal reclamation, the local economy and the marine ecological environment. The results are shown in Fig. 2.

The proportions of water quality data for Grade IV and Inferior to Grade IV (GB3097-1997) were calculated to show the pollution degree of sea water^21^.

According to the correlation analysis, the coastal reclamation area was significantly correlated (Fig. 2a) with the growth of GDP at the p < 0.01 level from 1995 to 2015 in the TBNA, with a coefficient of 0.704 (indicating a moderate relationship). The coastal reclamation area and pollution degree of sea water represented no significant relationship with a coefficient of 0.421 (Fig. 2b). While when correlation analysis tried with the data point of 2009 excluded (Fig. 2c), it was found that the variables were significantly correlated at the p < 0.01 level, and the coefficient 0.726 suggests a moderate relationship.

The above results suggest that an increase in coastal reclamation activities had a positive impact on the local economy. Coastal reclamation and sea water quality also showed a negative correlation when the data point of 2009 was excluded.

Figure 2a shows that when the coastal reclamation area is less than 100 km^2^, the discrete points show a strong linear relationship but when the coastal reclamation area is more than 100 km^2^, the discrete points are scattered, and the linear relationship is weak. However, the trend of this kind of change can only be qualitatively judged from the correlation coefficients, and the time series cannot be used to quantitatively show the relationship between the coastal reclamation area and the increment of GDP. Therefore, the decoupling elasticity values method was used to analyze the trend of the relationship in time series.

### Results of decoupling analysis

**Figure 3:**
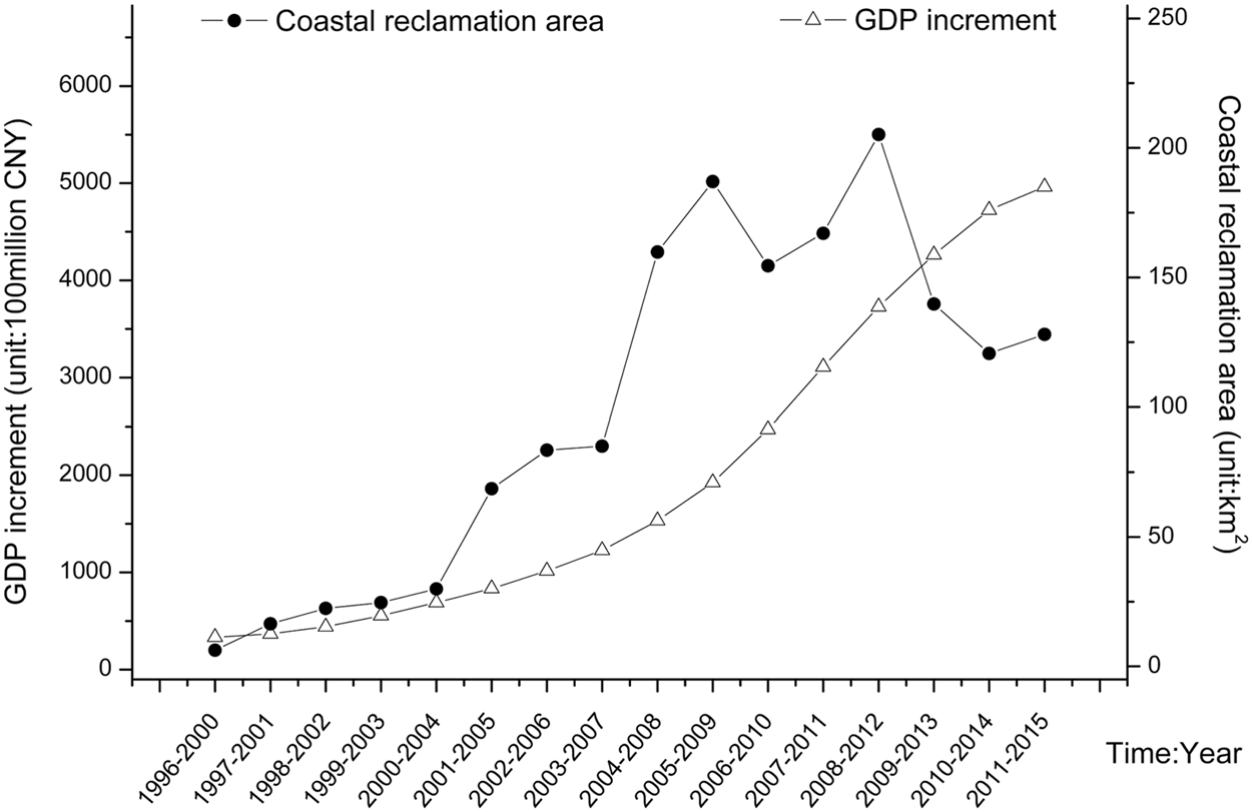
Graph of coastal reclamation area and GDP growth.

**Figure 4:**
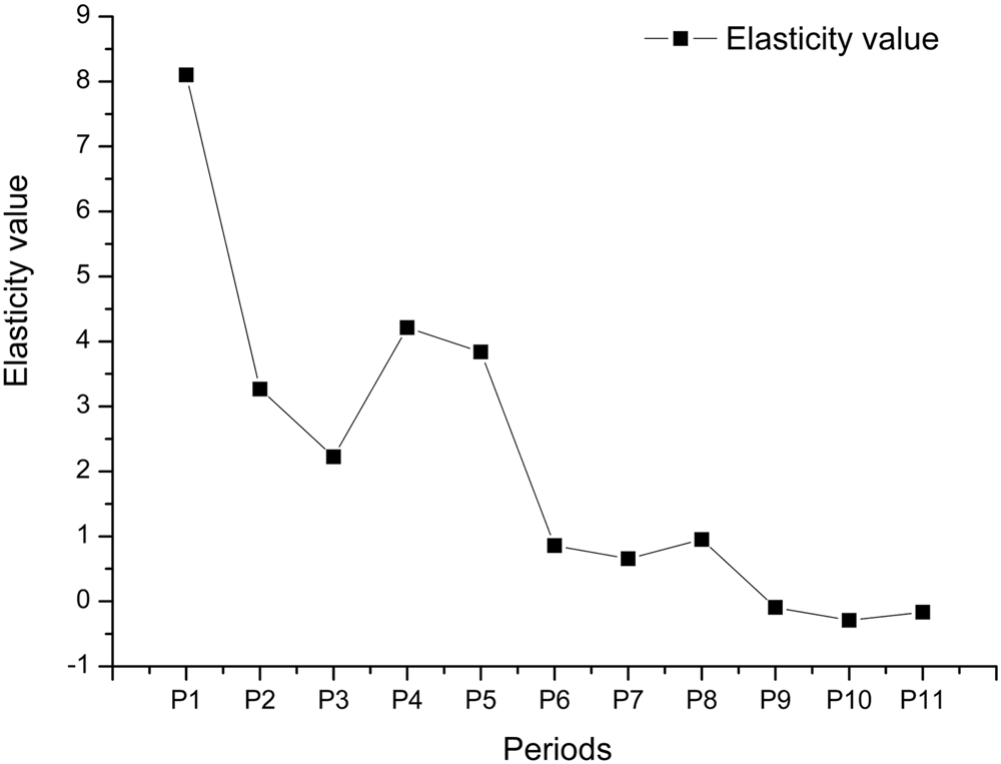
Change in elasticity value between coastal reclamation area and GDP with time.

Changes in GDP and total coastal reclamation area during every period are shown in Fig. 3. Growth in GDP shows a steadily increasing trend, while the growth in area of coastal reclamation has fluctuated over the last six periods. Based on equation 2, the rate of change of coastal reclamation and GDP growth were used to measure the elasticity value and types of decoupling, which are shown in Table 2. The cumulative trend in elasticity values is shown in Fig. 4.

**Table 2:** Table of the decoupling model.

Period	Change rate of coastal reclamation (%)	GDP change rate (%)	Elasticity value	Type of decoupling
**P1(2001–2005)**	**998.07**	**123.21**	**8.10**	**Expansive negative decoupling**
P2(2002–2006)	408.50	125.01	3.27	Expansive negative decoupling
P3(2003–2007)	278.87	125.39	2.22	Expansive negative decoupling
P4(2004–2008)	549.34	130.39	4.21	Expansive negative decoupling
P5(2005–2009)	524.83	136.75	3.84	Expansive negative decoupling
**P6(2006–2010)**	**125.09**	**145.98**	**0.86**	**Expansive coupling**
P7(2007–2011)	99.96	152.46	0.66	Weak decoupling
P8(2008–2012)	141.35	148.48	0.95	Expansive coupling
P9(2009–2013)	−12.57	137.50	−0.09	Strong decoupling
P10(2010–2014)	−35.47	121.77	−0.29	Strong decoupling
**P11(2011–2015)**	**−17.16**	**103.34**	**−0.17**	**Strong decoupling**

Note: Data in the three main periods (e.g. 2001–2005, 2006–2010 and 2011–2015) are shown in bold for the convenience of identifying decoupling change trends in the whole time series.

There were 11 periods altogether in the study (Fig. 4), with three main periods P1 (2001–2005), P6 (2006–2010) and P11 (2011–2015) covering the whole time series and some transitional periods.

In P1, the growth rate of coastal reclamation and GDP showed extensive negative decoupling correlations with an elasticity value of 8.1, which means that an increase in GDP should be accompanied by a very large increase in coastal reclamation. It also indicates that an increase in coastal reclamation had a weak effect on raising the GDP growth rate. Table 2 and Fig. 4 show that extensive negative decoupling continued during the following four periods (from P2 to P5), with the elasticity value dropping from 8.1 to 2.22 and then rising to 3.84. Overall, the dependence of GDP growth on the rate of coastal reclamation was gradually strengthened during those time periods.

In P6, the growth rate of coastal reclamation and GDP reflected an extensive coupling with an elasticity value of 0.86, indicating that coastal reclamation and GDP grew at almost the same rate. This shows that the growth in coastal reclamation area had a significant effect on increasing the growth of GDP, with a close correlation. During the following two periods (P7 and P8), the decoupling type changed from extensive coupling to weak decoupling and back to extensive coupling, which means that GDP growth rate continued to be related to and dependent on the growth of coastal reclamation.

P11 continued the trends of P9 and P10 to show a strong decoupling state with a negative elasticity value, which means that the GDP growth rate was increasing while the rate of coastal reclamation was decreasing. During this time, the growth rate of GDP was independent of the growth rate of coastal reclamation.

## Discussion

### The effects of coastal reclamation on the growth of the economy

According to the correlation analysis, the coastal reclamation area was significantly correlated with the growth of GDP. Human activities have resulted in the coastline of the TBNA continually extending seawards during the period from 1996 to 2015, especially after the TBNA was included in the national overall development strategy. Expansion of construction land and wetlands increased the overall length of the coastline from 145.48 km to 319.24 km, and the area of construction land increased by 227.05 km^2^. GDP growth is up to 4963.02 billion CNY during 2011–2015, more than five times the amount of growth during 2001–2005, and the economy expanded rapidly.

High-intensity coastal reclamation was mainly driven by the booming economy, especially after 2000, associated with urbanization and industrial development in China’s coastal region^8^. With rapid economic development and increasing population in coastal areas, reclamation has been regarded as an effective measure to resolve the land shortage as cities and industries expand^29^. Tian *et al*.’s analysis also indicated that all of the coastal provinces with high-intensity reclamation showed the same trend of GDP per capita being highly related to coastal reclamation^8^. In turn, extensive coastal reclamation led to economic growth. Worldwide reclamation in the past often incurred relatively low cost but harvested huge profit^30^. The situation is similar in TBNA, where reclamation land is focused on the development of port and coastal industries. In the construction of the Tianjin New Port, the land price amounted to about 2.50 times of the costs of engineering and sea-use fee for the reclamation^31^.

Coupling analysis showed that the dependence of GDP growth on coastal reclamation fluctuated in the time series. Before 2005, coastal reclamation in the TBNA was rapid^32^ with no long-term, comprehensive and reasonable planning of coastal reclamation projects. In addition, there were problems with water safety during this stage because of inadequate approval systems and procedures. Therefore, although the reclamation activities had a great impact on the ecological environment, their promotion of economic growth was not obvious. In 2005, the TBNA was incorporated into the national overall development strategy, and in 2006 the national reclamation plan was implemented. Tianjin began to invest in the construction of coastal reclamation projects. In July 2006, the construction of the first harbor at Dongjiang port had completed one third of the total coastal reclamation work of the Tianjin port construction. By 2009, the Nangang industrial zone had reclaimed 45 km^2^ of coastal land. During this period, with the support of the national reclamation policy, the coastal reclamation activities in Tianjin increased rapidly. At the same time, the economy grew rapidly at a rate of 130.96%. The dependence of economic growth on coastal reclamation was gradually strengthened. The decoupling elasticity value decreased as a whole, getting closer to the coupling state. According to the reclamation plan for Tianjin port, most of the reclamation work was completed by 2012. Some infrastructure projects on the reclaimed land have been completed and put into operation. The role played by coastal reclamation in economic growth is expected to gradually reduce.

By 2015, the decoupling state had become strong decoupling, with almost no dependence of GDP growth on the rate of reclamation. Economic development began to rely on other industries and required less additional land.

### The effects of coastal reclamation on the quality of inshore seawater

In 2009, seawater was slightly polluted, with only 30% of Grade IV and Inferior to Grade IV, a break in the time series which was somewhat unexpected. Based on Xu *et al*.’s research^33^, dissolved inorganic nitrogen and dissolved inorganic phosphorus are the main pollutants in TBNA, and the great change in the water quality in 2009 was due to the reduction of dissolved inorganic nitrogen in the sea water. Growing runoff into the Sea is the main cause of the rapid increase of dissolved inorganic nitrogen^33^. While, in 2009, total precipitation days and heavy rain decreased significantly, leading to less runoff into the sea compared with the other years during the time series^34^, which explains why higher water quality occurs in 2009. Once the results from 2009 were excluded, there was a significant correlation between the two and the analysis was in line with the actual situation. Therefore, these adjusted data are used in the discussion and conclusion.

The growth in coastal reclamation in the TBNA has brought about a decline in the quality of coastal seawater. Result indicates that the water quality of Tianjin’s inshore water is gradually deteriorating under the influence of intense coastal reclamation. Before 2009, the proportion of Grade IV and Inferior to Grade IV seawater for most of the years was under or around 50%, showing moderate pollution. After 2009, the proportion of Grade IV and Inferior to Grade IV seawater was higher than 50% in each year, showing that seawater was severely polluted. From an environmental point of view, instances of water quality of Grade IV and Inferior to Grade IV are gradually increasing, and the deteriorating seawater quality will have a negative impact on the marine ecological environment.

With the impetus of national policy, reclamation activities in the TBNA are continuing to intensify, leading to the development of the local economy and an improvement in the living standards of local people. However, rapid and large-scale reclamation activities presently carried out in China’s coastal areas have far exceeded the carrying capacity of the natural environment^29^. The advanced sand blowing technology makes it possible to reclaim 1 km^2^ of land from the sea in about 20 days^29^. Land reclamation would reduce water purification ability from narrowing and even disappearance of gulfs and bays, increase water pollution and frequent harmful algal blooms, producing great damage to the quality of near shore sea water^8, 35^.

Overall, coastal reclamation activities had a serious impact on the inshore seawater quality. Because of the influence of pollutants from the construction of industrial zones and port operation, this is likely to have cumulative effects on the ecological environment^23^. In view of current state of water quality, we predict that the negative influence of coastal reclamation on ecological environment will continue, leading to an on-going deterioration in water quality.

### Coordinated development between coastal reclamation, the economy and the ecological environment

The implementation of coastal reclamation projects in the TBNA has brought great benefits for the economic development. However, the benefits are gradually weakening as the projects are completed, while the quality of the ecological environment continues to deteriorate. In the process of coastal reclamation project, the balance of reclamation, economic development and environmental protection is poor, leading to a degraded ecological environment. Activities such as the construction of industrial infrastructure and the expansion of urban areas will take place on the reclaimed lands, and coastal reclamation will become a group of highly intensive activities^8, 23, 29^. If development is at the expense of the environment, the overall benefit of reclamation will be greatly reduced. The rapid expansion of artificial coastlines to seaward would weak sediment dynamics and hydrodynamics, decrease the biodiversity^23^ and intertidal habitats as to lower the ecosystem services^25^, leading to the reducing of the overall benefit of coastal reclamation^25, 36^. Sustainable development should consider all three factors, environmental sustainability, economic sustainability, and social sustainability^17^. Changes in quality or quantity of ecosystem services either change the benefits associated with human activities or alter the costs of maintaining human welfare. Because ecosystem services are largely outside the market and uncertain, they are too often ignored or undervalued, leading to the error of constructing projects whose social costs far out weight their benefits^17^. Effective planning and management are preconditions for sustainable coastal development^37^.

Therefore, to ensure that sustainable development balances all three aspects, some measures should be taken in coastal reclamation. First, more effective regulation measures in line with the city’s economic and environmental development should be taken to avoid unsustainable reclamation projects. During the time series, sea water pollution degree is significantly correlated with the area of reclamation, which means that sea reclamation still contributes to the deterioration of sea water quality, while the contribution of sea reclamation to economic benefit began to decline with a strong decoupling state. At present, China’s understanding of coastal reclamation is in transition from the warm expectation stage to the rational and prudent stage, but coastal reclamation is still the first choice when settling the conflict between human and land resources. This emphasizes the need for comprehensive and more effective regulation measures for coastal reclamation. We must evaluate the pros and cons of economic development and environment protection, and then carry out reasonable coastal reclamation activities to maintain the sustainability of regional economic development. Second, in the present reclamation areas, environmental deficiencies must be rectified so that the projects become more sustainable in the operational phase. Third, the legal basis of coastal reclamation needs to be improved. The management of reclamation in China is mainly based on the Sea Use Management Law (released in 2002), which involves the application and approval of the sea area and the right to use the sea area. More detailed constraints on coastal reclamation should be clearly established. Management of anthropogenic disturbances to artificial habitat is necessary. Economic quantification of lost ecosystem services should be added into the decision making process. Increasing our understanding of the ecological functioning of marine habitats created by urban infrastructure and incorporating ecological criteria into coastal engineering are crucial for preserving biodiversity^24^. Achieving this goal will need strong collaboration between engineers, managers and ecologists.

There are several limitations associated with the research presented here. The justification of the removal of data point of 2009 was deduced from former literature, and no direct data were achieved to explain it, which may lead to some inaccuracies. In the present research, only the effects of land reclamation on economy and water quality were analyzed, while the relationship between GDP and water quality was not involved, which would be carried out in the future research. From the aspect of analysis methods, owing to the limit of data source, water quality can be achieved only from 2001, so the decoupling elasticity model was not used to test reclamation with water quality in this study.

## Conclusions

Based on remote sensing data from Landsat TM/ETM+/OLI, this paper quantified the coastline change of the TBNA from 1995 to 2015 and then used correlation analysis to analyze the relationship between coastal reclamation and GDP growth and between coastal reclamation and seawater quality. Finally, a decoupling elasticity value model was used to study the dependence of economy growth rate on the rate of coastal reclamation during different time stages and the reasons of the dependence were analyzed. The results show that:

1. Over time, the artificial coastline of the TBNA showed an increasing trend. Before 2005, the coastline had a slow growth but from 2005 to 2010, the coastline grew rapidly. By 2015, the growth rate of the coastline decreased but the growth was still large. The major cause of coastline growth was human coastal reclamation activities, and the main types of reclaimed land were construction land and wetlands.
2. Over the time series, the coastal reclamation area and GDP growth were moderately related with a correlation coefficient of 0.704 (significant at p < 0.01). Having removed the outlying data from 2009, coastal reclamation area and water pollution degree also showed a moderate correlation with a correlation coefficient of 0.726 (significant at p < 0.01). This showed that coastal reclamation has obvious influence on economic development and environmental degradation.
3. According to the decoupling analysis, the decoupling relationship changed from expansive negative decoupling in P1 to weak decoupling in P6 to strong decoupling in P11. During 2001–2005, the dependence gradually strengthened, and from 2006 to 2010, GDP growth rate remained related to the growth of coastal reclamation area. Since 2011, the GDP growth rate has become independent of the growth rate of coastal reclamation. Overall, the dependence of economic growth on coastal reclamation has gradually weakened.

Comprehensive analysis showed that coastal reclamation activities in the TBNA have played an important role in promoting economic development, but at the same time, they have degraded the ecological environment. Although the promotion has weakened over time, the influence on the inshore water quality will continue because of the cumulative effect of pollution. To keep the relationship between coastal reclamation, economic development and environmental protection in balance, this paper recommends that 1) planning coastal reclamation must address both economic and environmental outcomes; 2) environmental deficiencies from existing coastal reclamation projects must be rectified; and 3) the legal system of coastal reclamation must be improved.

## Materials and Methods

### Study area

**Figure 5:**
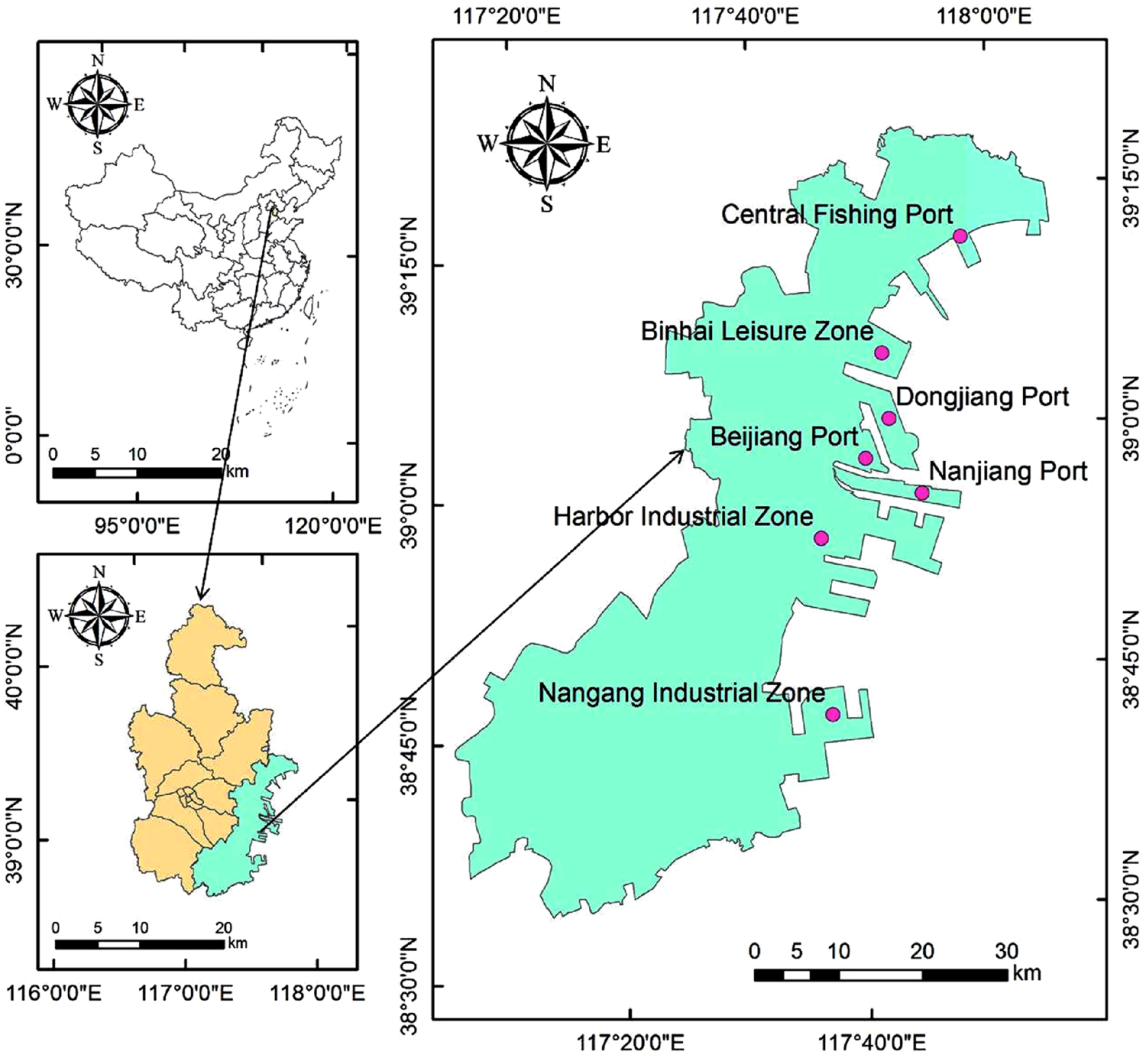
Location of Tianjin Binhai New Area. Figure 5 was generated by ArcGIS v10.2 software (Environmental Systems Research Institute, Inc, USA, URL http://www.esri.com/).

The TBNA is located on the east coast of Bohai Bay, at the junction of the Shandong Peninsula and Liaodong Peninsula between 38°40′39°00′N and 117°20′–118°00′E (Fig. 5). It lies east of Tianjin city center, at the mouth of the Haihe River and covers a land area of 2,270 km^2^ and a sea area of 3,000 km^2^. The landform is a coastal alluvial plain, higher in the northwest and lower in the southeast with altitudes between 1–3 m.

The TBNA has a continental monsoon climate with some oceanic climate characteristics. The average annual temperature is 12.3 °C, with the highest and lowest recorded temperatures being 40.9 °C and −18.3 °C respectively. The precipitation changes with the season and concentrates in summer, with a mean annual precipitation of 566 mm. Some meteorological extremes such as gales, heavy fog, rainstorms, storm surges and dust storms occur in the area.

The TBNA has more than 700 km^2^ of water and wetland combined, including beach swamps, coastal beaches, estuarine waters and other natural wetlands and salt ponds, reservoirs, rice fields and other wetlands. Tianjin is planned to be an international port city. The construction of Tianjin port is still in progress and coastal reclamation is the main way to provide land resources for construction.

National policy has led to the rapid growth of the economy of the TBNA, especially in recent years with the integration of Beijing, Tianjin and Hebei. Over this time, human activities in the TBNA have had a great impact on the ecological environment^38^. Near-shore seawater has become polluted and the quality has continued to worsen, with main pollutants being inorganic nitrogen and active phosphate^39^.

### Data acquisition and preprocessing

Coastline extraction was mainly based on Landsat TM/ETM + /OLI imagery from 1995 to 2015, which were downloaded from the Geographic Spatial Data Cloud website (http://www.gscloud.cn/) and USGS website (http://glovis.usgs.gov/). The acquired time of downloaded image data were all from July to September to keep the interpretation results comparable between all these years. Band fusion, image correction, image enhancement and color blending were used to generate multi-spectral color remote sensing images with a spatial resolution of 30 m (TM) and 15 m (ETM+/OLI).

Sea water quality data were acquired from “China’s coastal Marine environment quality bulletin” (for each year from 2001 to 2014), which were downloaded from the Chinese Environment Monitoring Station (http://www.cnemc.cn/), where sea water quality is defined based on “Sea water quality standard” (GB3097-1997). 35 index (including pH value, inorganic nitrogen, dissolved oxygen, chemical oxygen demand (COD), petroleum, active phosphate, etc.) are utilized to make the definition of the basic four quality grades by single factor discriminant method. When any of the evaluation index of seawater exceeds the standard of Grade I, it is defined as Grade II, and so on. When the quality of sea water could not meet the standard of Grade IV, then it was defined as Inferior to Grade IV. Water quality data for Grade IV and Inferior to Grade IV (GB3097-1997) were collected for the TBNA during 2001 to 2014 and their proportions were calculated to show the sea water pollution status^40^, shown as “pollution degree of sea water” in this paper.

GDP (Gross Domestic Product) data for the TBNA from 1995 to 2015 were taken from the statistical yearbook of Tianjin (http://www.stats-tj.gov.cn/Article/List/List_29.html), and GDP values at a comparable price were calculated as a measure of the economic situation of the study area.

### Coastline extraction and reclamation statistics

Computer-based automatic interpretation, together with visual interpretation, was applied to extract the coastline length and the area of coastal reclamation. The Normalized Difference Water Index (NDWI) can be used for the extraction of coastlines^41^ and Xu^42^ improved the index by creating a Modified NDWI (MNDWI) to obtain higher interpretation accuracy. In the calculation of MNDWI, the near infrared band is replaced by the mid infrared (MIR) band, thus the influence of buildings and soil on the coastline extraction is reduced. The accuracy of the coastline extraction method based on MNDWI is higher than using NDWI. Therefore, MNDWI was adopted in this paper. The equation of MNDWI is as follows:

1 $$MNDWI=\frac{Green-MIR}{Green+MIR}$$

Green and MIR represent the green band and mid infrared band, respectively.

The classification results were tested with real monitoring samples and Google Earth. About 200 ground truth points were acquired for the accuracy assessment through field surveys and querying in Google Earth. Based on these ground truth points, unified rectification was carried out to enhance the accuracy of classification.

### Correlation analysis

In this paper, correlation analyses were carried out with SPSS 19.0 software. The Pearson coefficient of product-moment correlation was calculated and a t-test was used to verify the results. The correlation results were classified into four grades according to the coefficients, weakly related or not related ([0, 0.3]), represents low related ([0.3, 0.5]), medium related ([0.5, 0.8]) and high related ([0.8, 1.0]).

Because of the complex changes in the coastline, the impacts of change on the environment exhibit hysteresis. Through the comparative analysis based on the data in the study area, it was found that the cumulative effect of coastal reclamation is more significant within 5 years. Therefore, a 5-year step-length was used here to summarize the total area of coastal reclamation and the total value of GDP over the 5 years. Taking every year as a time node, a total of 16 sets of research data were achieved (e.g., 1996–2000, 1997–2001 … 2011–2015). Similarly, the time series data of annual pollution degree of sea water were correlated with the series data of 5-year total coastal reclamation area due to the cumulative effects on the ecological environment. Owing to the limits of data acquisition, the analysis period is focused on the period of 2001–2014.

### Decoupling analysis

Decoupling analysis has been used by many scholars to study the relationship between environmental pressure and economic growth^43–47^. Both the decoupling indicators of the OECD’s model^48, 49^ and the decoupling elasticity value of Tapio’s model^50^ have been used in these studies. However, the OECD’s decoupling indicator model can only qualitatively judge if variables are coupled or decoupled, with no decoupling degree provided. Tapio’s decoupling elasticity value model can classify the decoupling relationship into eight types according to the decoupling degree. This shows the decoupling relationship and its variation more clearly. In this paper, therefore, Tapio’s decoupling elasticity value model was used to study the decoupling relationship and variation between coastal reclamation and economy development in the TBNA.

In Tapio’s model, the decoupling elasticity value indicates the change rate of A divided by the change rate of B in a given time period to show the decoupling relationship between A and B. Here, a decoupling elasticity model was constructed for the decoupling of the change rate of the area of coastal reclamation (*%ΔCR*) from the growth rate of *GDP* (*%ΔGDP*) (equation 2).

2 $${\beta }_{i}=\frac{ \% {\rm{\Delta }}CR}{ \% {\rm{\Delta }}GDP}=\frac{(C{R}_{pi}-C{R}_{former})/C{R}_{former}}{(GD{P}_{E}-GD{P}_{LS})/GD{P}_{S}}$$

*β*_*i*_ is the decoupling elasticity value (decoupling degree) of the *i*th period P*i*, showing *GDP* elasticity of *CR*. Because of the cumulative effect of coastal reclamation, the 5-year step-length was still used in the decoupling analysis. *CR* is itself a variable quantity, so *ΔCR* was calculated as the change rate of *CR* in the present period (*CR*_*pi*_) compared with that in the former period of 5 years (*CR*_*former*_). Thus, starting from 2001, altogether 11 periods were used (2001–2005, 2002–2006 … 2011–2015).

**Figure 6:**
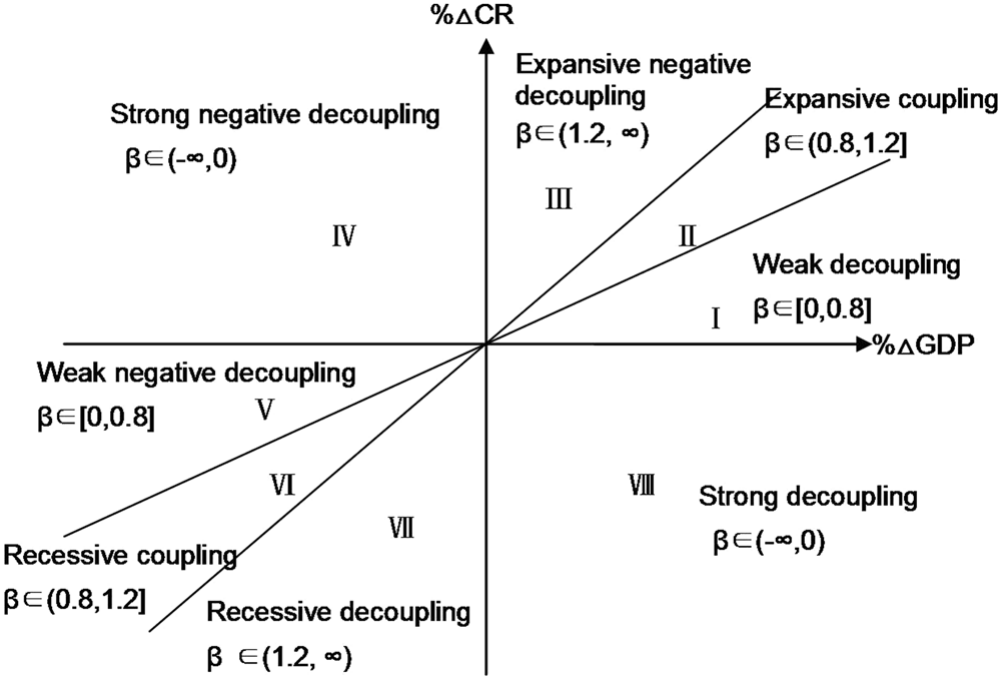
Coordinated figure of decoupling types (modified from Tapio)^39^.

The classification and principle of the decoupling state are shown in Fig. 6 ^48, 50–52^.

Eight logical possibilities can be distinguished (Fig. 6) ^39^. Results can be coupled (expansive coupling and recessive coupling), decoupled (weak decoupling, strong decoupling and recessive decoupling) or negatively decoupled (expansive negative decoupling, weak negative decoupling and strong negative decoupling).

An elasticity value of between 0.8 and 1.2 indicates a coupled state, which can be further divided into two parts: expansive coupling (section II in Fig. 6) and recessive coupling (section VI in Fig. 6). Expansive coupling indicates that coastal reclamation makes a great contribution to GDP growth, and the growth of GDP runs in tandem with an increase in coastal reclamation. Recessive coupling means that a decrease in GDP growth is related to a reduction in coastal reclamation.

Decoupling can be divided into three subcategories: weak decoupling (β ∈ (0, 0, 8)) indicates that coastal reclamation makes a small contribution to unit GDP growth [part I, Fig. 6]; strong decoupling (β ∈ (−∞, 0)) [part IV, Fig. 6] shows that regional economic development does not depend on the coastal reclamation activities, which if environmental degradation is high due to land reclamation, that reclamation should not be justified based on economic purposes; recessive decoupling (β ∈ [1.2, +∞]) indicates that a decrease in the unit GDP increment is accompanied by a greater decrease in the area of coastal reclamation [part VII, Fig. 6].

Similarly, negative decoupling includes three subcategories: expansive negative decoupling (β ∈ [1.2, +∞]) indicates that every unit of GDP growth requires a substantial increase in coastal reclamation [part III, Fig. 6], while weak negative decoupling (β ∈ (0, 0, 8)) indicates that a decrease in unit GDP growth is partly caused by a reduction in coastal reclamation [part V, Fig. 6]. When ∆CR > 0 and ∆GDP < 0, it represents strong negative decoupling [part IV, Fig. 6]. In this case, increasing coastal reclamation activities does not promote economic growth and the local economy declines. This is the most unsatisfactory outcome.
